# COPO - Managing sample metadata for biodiversity: considerations from the Darwin Tree of Life project

**DOI:** 10.12688/wellcomeopenres.18499.2

**Published:** 2023-07-31

**Authors:** Felix Shaw, Alice Minotto, Seanna McTaggart, Aaliyah Providence, Peter Harrison, Joana Paupério, Jeena Rajan, Josephine Burgin, Guy Cochrane, Estelle Kilias, Mara K.N. Lawniczak, Robert Davey

**Affiliations:** 1 Earlham Institute, Norwich, Norfolk, NR4 7UH, UK; 2 EMBL European Bioinformatics Institute, Hinxton, Cambridgeshire, CB10 1SD, UK; 3 Department of Zoology, University of Oxford, Oxford, Oxfordshire, OX1 2JD, UK; 4 Wellcome Trust Sanger Institute, Hinxton, Cambridgeshire, CB10 1RQ, UK

**Keywords:** biodiversity, standards, sharing, metadata, samples, Darwin Tree of Life, taxonomic domains, data management, LIMS, metadata standards

## Abstract

Large-scale reference genome sequencing projects for all of biodiversity are underway and common standards have been in place for some years to enable the understanding and sharing of sequence data. However, the metadata that describes the collection, processing and management of samples, and link to the associated sequencing and genome data, are not yet adequately developed and standardised for these projects. At the time of writing, the Darwin Tree of Life (DToL) Project is over two years into its ten-year ambition to sequence all described eukaryotic species in Britain and Ireland. We have sought consensus from a wide range of scientists across taxonomic domains to determine the minimal set of metadata that we collectively deem as critically important to accompany each sequenced specimen. These metadata are made available throughout the subsequent laboratory processes, and once collected, need to be adequately managed to fulfil the requirements of good data management practice.

Due to the size and scale of management required, software tools are needed. These tools need to implement rigorous development pathways and change management procedures to ensure that effective research data management of key project and sample metadata is maintained. Tracking of sample properties through the sequencing process is handled by Lab Information Management Systems (LIMS), so publication of the sequenced data is achieved via technical integration of LIMS and data management tools.

Discussions with community members on how metadata standards need to be managed within large-scale programmes is a priority in the planning process. Here we report on the standards we developed with respect to a robust and reusable mechanism of metadata collection, in the hopes that other projects forthcoming or underway will adopt these practices for metadata.

## Introduction

Datasets are fundamental assets in the life sciences, where knowledge is routinely extracted and interpreted on the genomic scale ( International Society for Biocuration, 2018). The robustness of sequencing experiments relies on the accuracy of measurements regarding accompanying sample observations. Much effort goes into the production and validation of methods and models for data, with mechanisms for sharing this information being largely standardised. For example, consensus was quickly reached for including quality information into the file format used for sharing sequence data (FASTQ). Where human record-keeping is the deciding factor in the quality of information, there has been far less formal standardisation. The field of biocuration is an essential part of the research data lifecycle in order to cope with the quantities and complexities of modern data generation ( Howe *et al.*, 2008). However, it can be an expensive and onerous process to produce, implement and adopt such a standard within a community, to apply *post hoc* curation, and to provide associated data storage and tools ( Kennan & Markauskaite, 2015), ( Whitmire *et al.*, 2015) ( ten Hoopen *et al.*, 2016).

For software, data not provided in the correct format can cause runtime errors and erroneous results. Incorrect or absent metadata, though, may not necessarily break a system in the same way, but accurate metadata is a vital layer of context providing understanding for humans, and interoperability for machines. Therefore, metadata standardisation is equally as important as data standardisation. Still, there is often no requirement for tools, software, and analytical processes to check metadata quality, and little support given to scientists who have to carry out data curation.

The DToL project oversees the collection of diverse species from a wide range of habitats with the goal of producing draft genomes that are immediately available to the community. Recent articles have highlighted the importance of standardisation and formed a concise set of rules for researchers to follow in their sequencing experiments ( Stevens *et al.*, 2020). Ensuring that future studies based on DToL reference genomes (such as resequencing), can be contextualised against a range of observable variables is essential. Therefore standardised and accurate recording of properties such as identifiers, taxonomic information, lifestage, body parts, labs and organisations involved, collectors and collection events, collection environments, identification uncertainties, hazard groups, preservation, barcoding, regulatory compliance and vouchering is key. In our sister paper, we describe the processes and procedures set up by DToL to ensure consistency and standardisation of metadata across the project ( Lawniczak *et al.*, 2022).

Briefly, DToL samples are collected by and processed within the oversight of a Genome Acquisition Laboratory (GAL). Taxonomic experts who have in-depth knowledge of their research organisms, their respective habitats and/or historical collections, prepare the original specimen into sequencing-ready material. Whilst this may involve different laboratory processes, the same endpoint must be reached. A sample must be collected with the properties required for sequencing at the required depth and coverage to produce a reference quality genome. To do so, the DToL project set up a Samples Working Group to bring together researchers representing all GALs as well as six different eukaryotic taxonomic areas. These are plants, arthropods, lichens and fungi, Chordata, Protista, microalgae and other Metazoa (mainly comprising non-arthropod invertebrates). The members of the Samples Working Group meet twice monthly and are tasked with developing the target list of priority species, standardising metadata collection for the project, and developing, refining, and standardising collection procedures for these different taxonomic groups. This group also comprises researchers who will receive these physical samples for subsequent preparation into sequencing libraries, and is charged with developing protocols for sample storage, delivery, and compliance.

## Sample manifest development

A project of the scale and ambition of DToL calls for a substantial effort in developing, monitoring and refining its metadata collection and management infrastructure. We informed our strategy through the alignment with existing specifications in the biodiversity domain, such as the Biodiversity Information Standards (formerly Taxonomic Databases Working Group, TDWG) Darwin Core. Bringing together past experiences in developing and implementing metadata standards from the Wellcome Trust (WT) Sanger, Collaborative OPen Omics (COPO) and European Nucleotide Archive (ENA) teams, in particular community-led collaborative work on such standards as MiXS ( Yilmaz *et al.*, 2011) and CG Core ( *GitHub - AgriculturalSemantics/cg-core: CG Core Metadata Reference Guide*, 2021)), the DToL team focused its efforts into its sample manifest infrastructure.

As outlined in our recent article ( Lawniczak *et al.*, 2022), the DToL specimen collectors need to record information associated with the location and time a specimen was taken for the project. Each specimen is required to represent (if possible) a single genetic entity or individual, and digital identification of these specimens and subsequent samples must be accurately represented in data management tools. Multiple samples may be taken from the same specimen ( *e.g.* different tissues from a large animal are put into different tubes) and this information must be tracked (see “Relationships between samples” below). Each tube containing a sample is assigned a SPECIMEN_ID, which is a unique identifier generated by the GAL, intending to reflect the genetic identity of the organism contained within it; symbionts, contamination and co-occurring cultures notwithstanding. Tubes also get a TUBE_OR_WELL_ID, which is the barcode stamped on the tube and represents a sample of that specimen. Some organisms are too small to be collected in tubes and this is reflected in the name “TUBE_OR_WELL_ID”. This also gives future scope for the more widespread collection and processing of samples in plates and wells. In either case, each individual sample can be identified by the concatenation of RACK_OR_PLATE_ID and TUBE_OR_WELL_ID. When two or more samples are taken from the same specimen ( *e.g.* insect blood in one tube, a leg in another), any difference between these is captured in further fields. Cultured protists are presumed to be processed in the same way as other environmental samples. In this case, an ENV_SAMPLE_ID will be assigned to the sample, and this identifier will also be referred to in the derived single cell samples together with the SPECIMEN_ID. Unculturable protists and other organisms may be collected for both metagenomic analysis and single cell sequencing.

The DToL Samples Working Group identified the initial information that would describe the sample collection events to form a *sample manifest* specification. The sample manifest needed to be sufficient to cover common metadata across all taxonomic groups. This resulted in a single core schema document that comprises the set of metadata fields that need to be provided by a GAL before the sample material is accepted for sequencing ( Lawniczak *et al.*, 2022). In the DToL project, the sample manifest is the initiating step in the tracking of the sample from collection to sequencing to release of the data in the public repositories ( Cummins *et al.*, 2022) and further display on the DToL Data Portal ( *Darwin Tree of Life*, 2022). In this way, the DToL sample manifest is suitable as a starting point for use in programmes associated with the Earth Biogenome Project, and potentially others, which will require significant organisation and collaboration to ensure comparison and reuse of the vast quantities of data that will be produced in the coming years.

To promote transparency, openness and to enable controlled versioning of the standards, the core manifest and standard operating procedure (SOP) are publicly available from the DToL GitHub repository.

When there are metadata divergences depending on the taxonomic groups, for example where samples are collected in wells or tubes depending on their physical size, these can often be mitigated by common fields. For example, we use PLATE_OR_RACK_ID and TUBE_OR_WELL_ID, respectively, to ensure that we have a consistent identification strategy.

In other cases where different fields are required for accurate metadata modelling, the DToL manifests can act as a basis to develop “extension” manifests to cover metadata fields that are specific to a single taxonomic group. For example, marine researchers may be concerned with the salinity, depth and pH of the seawater at the point a sample is collected, but avian researchers are unlikely to record these properties. Modelling these differences is important to meet community requirements in these varying scenarios, but it is equally important to have a firm idea of core fields that will be appropriate for all scenarios.

The workflow of agreed changes via the DToL Samples Working Group is documented in shared Google Documents, and the list of proposed changes is placed as a link to a separate document in the SOP. After the initial phase of the project it was agreed the manifest would be updated twice a year. This is both because the list of agreed metadata to collect is expected to become more stable with the project reaching maturity, and because a number of services need to implement and deploy changes in concert with each other. Depending on the type of changes required, updates can take from a few days for simpler issues to over a month for more complex changes. This makes frequent updates of the SOP problematic to manage, develop and deploy. The SOPs and Manifests are versioned and published on GitHub ( *GitHub - darwintreeoflife/metadata, 2023*). When querying COPO for samples, the manifest version under which the samples were submitted is returned along with the sample metadata. This means users are able to determine which fields will be present. In some cases, samples may be retroactively updated in response to a manifest update, for example when there is a change of field name. However these types of changes are highly discouraged.

The Darwin Tree of Life project is affiliated with the Earth Biogenome Project ( Lewin *et al.*, 2022). Other EBP projects such as ASG (Aquatic Symbiosis Genomics project) and ERGA (European Reference Genome Atlas) are currently building on DToL developments and are basing SOPs and processes on the DToL manifest. This will require the addition or removal of fields and controlled vocabulary terms as necessary. In both cases, it was agreed to follow the same timeline and to keep up to date with DToL updates given the overlap between the projects. This makes maintenance of software tools which handle these data types significantly less burdensome.

Once a version has been agreed upon, both the SOP and the sample manifest are openly published and versioned within the DToL GitHub repository so that changes can be implemented. Once a change is accepted and made to the SOP and/or manifest, developers of downstream information systems are informed and work can begin. Frequent changes include modification of column headers, addition of controlled vocabulary fields, and changing validation code so that verified submission to public repositories is maintained. The timeframe required by each type of change is approximately known so that there is a clear understanding of the process leading to a new release.

Where possible, we have mapped our manifest headers to TWDG Darwin Core terms in order to comply with the global standard for capturing occurrences and events around biodiversity monitoring. For example:

- DATE_OF_COLLECTION [ http://rs.tdwg.org/dwc/terms/verbatimEventDate]
- DECIMAL_LATITUDE [ http://rs.tdwg.org/dwc/terms/decimalLatitude]
- TUBE_OR_WELL_ID [ http://rs.tdwg.org/dwc/terms/measurementID]

Such mappings will allow us, in the future, to submit our records into these international databases and start to link genomic sequence information alongside sample metadata and images.

## EMBL-EBI ENA checklist

Data and associated metadata are archived in the ENA ( Cummins *et al.*, 2022,). There is a minimum amount of information required for registering sample metadata in the ENA and that is defined through the sample checklists. Different sample checklists have been developed to validate the requirements on the minimum metadata needed to describe biological samples submitted by different research communities. ENA staff work actively with members of these communities − often through such initiatives as the Genome Standards Consortium ( Field *et al.*, 2011) to capture appropriate requirements, understand working practices, and to integrate and/or map concepts across the different domains of life science. We produced an ENA sample checklist that reflects a subset of the DToL schema, called Tree of Life Checklist (ToL). The checklist was designed to validate all the metadata provided by the project which were deemed useful for public reuse and interpretation, and excludes metadata which are internal to project tracking. The checklist also aligns the metadata collected for the DToL manifest against other existing standards (for example the MIxS standards ( Yilmaz *et al.*, 2011)) to allow comparison with samples outside of the project. Both metadata and data can therefore be represented in this public repository in a standardised way. This allows all ToL datasets to be coherent across sample collection environments, sequencing protocols and machines, and scientific institutions. This also provides a minimal standard for any other datasets based on the DToL manifests which are not part of DToL, and form the backbone of other important international projects, *e.g.* ERGA and EBP. This is a significant contribution to open and FAIR scientific data, and we hope that the checklist will help other groups and programmes develop submission pathways that follow similar guidelines, improving reproducibility and consistency across a wider range of biodiversity projects.

## Metadata brokering

The timely and accurate submission of data and metadata to public repositories is a significant requirement for transparency in the life sciences ( Gonzalez & Peres-Neto, 2015), and the discipline of Research Data Management is gaining traction with funders, publishers, and researchers themselves ( Kennan & Markauskaite, 2015). However, researchers find it costly and difficult to transform information collected in the field or lab into consistent metadata that is suitable for meeting FAIR requirements ( Wilkinson *et al.*, 2016). The generic nature of existing submission routes where much of the metadata is not mandatory or unvalidated (somewhat mitigated by repository checklists) is one hurdle. That the submission systems themselves are not tailored to specific communities and therefore can be difficult to navigate for new users is another. COPO is a mature, actively developed web-based brokering system for annotating and depositing datasets to a number of public repositories ( Shaw *et al.*, 2020), and is able to be configured to fit with the needs of specific communities. COPO is used for brokering metadata between GALs and the ENA. Other services such as the LIMS at the Wellcome Sanger Institute pulls information from the COPO application programming interface (API) to insert into its own databases for downstream sample tracking in the lab. COPO is available for any researcher to use, but has also been developed to fulfil the needs of the DToL project, as well as other EBP-related projects such as ASG and ERGA. 

DToL users are assigned one of two groups in COPO. The Sample Submitters group is for users to upload their manifests, and this group is commonly made up of sample collectors themselves. The Sample Supervisors group is for users who will provide supervisory oversight and will accept or reject samples based on human curation post-validation, and these users often work at the GAL where the samples will be received. They will be presented with a view of all DToL samples needing approval. Rejected samples are held back within the system. If samples are accepted, they will be queued for submission to ENA, where they will be further validated against the ENA ToL checklist, and assigned ENA and BioSample accessions ( Courtot *et al.*, 2022).

DToL *Sample Supervisors* work with *Sample Submitters* and show them the sample manifest, assisting with any questions about its completion. The sample submitters/collectors fill in and submit the DToL Sample Manifest to COPO in the form of a tabular file, in Excel (CSV files are also accepted, but users prefer the more familiar and navigable format of spreadsheets). The file has many fields with drop down menus to help standardise spellings and terms as this is a common place for metadata tracking to diverge and can be difficult to resolve later. For example, collectors may refer to the sex of a specimen as F, f, Fem, FEMALE, etc. The use of data validation in the Excel helps avoid these issues for any term in which a relatively small set of defined entries is expected. We have used data validation for as many fields as possible, and the terms that are part of the subset of metadata submitted to ENA are mapped in the ENA ToL checklist.

When manifests are uploaded, they are checked for standards compliance to the SOP and multiple sets of validations are performed. Firstly, the manifest is validated against the NCBI Taxonomy for taxonomic integrity. Collectors must supply scientific names at the species level that match the main name in the taxonomy, and that are submittable to ENA (programmatic calls to the taxonomic query services at EBI return a “submittable” field). The taxonomic fields are then populated by COPO, as long as one of the fields SCIENTIFIC_NAME or TAXON_ID is provided, and synonyms are converted into main names with a warning to the user the main name will be stored instead.

**Figure 1.**
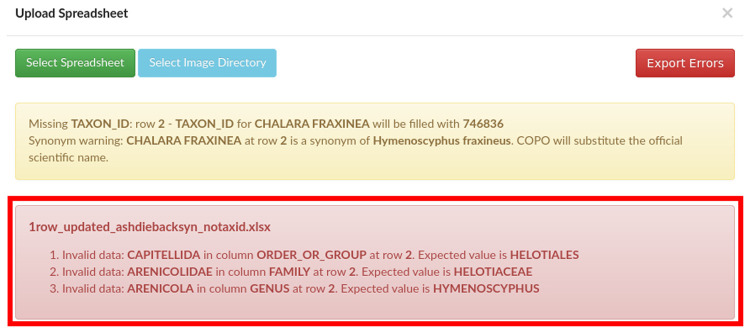
Collaborative OPen Omics (COPO) interface after an unsuccessful validation attempt, showing validation errors.

Provided the taxonomy validation passes, the manifest is then validated against the SOP specifications, whereby all mandatory values need to be present and formatted as described. As time has passed and a larger number of manifests have been submitted, we have included additional validation rules to mitigate against common human errors. A number of warnings are also shown in COPO where it is appropriate to invite the user to double check their entries (for instance for tube or well IDs which do not conform to the standard format). One such example of valuable validation are the checks for previous association of SPECIMEN_ID to a different TAXON_ID, and the trigger of an error if SPECIMEN_ID is found more than once when ORGANISM_PART is WHOLE_ORGANISM (as it would be impossible to have the entire individual collected in multiple tubes). Any validation errors are shown to the user (see Figure 1) and submission is aborted.

**Figure 2.**
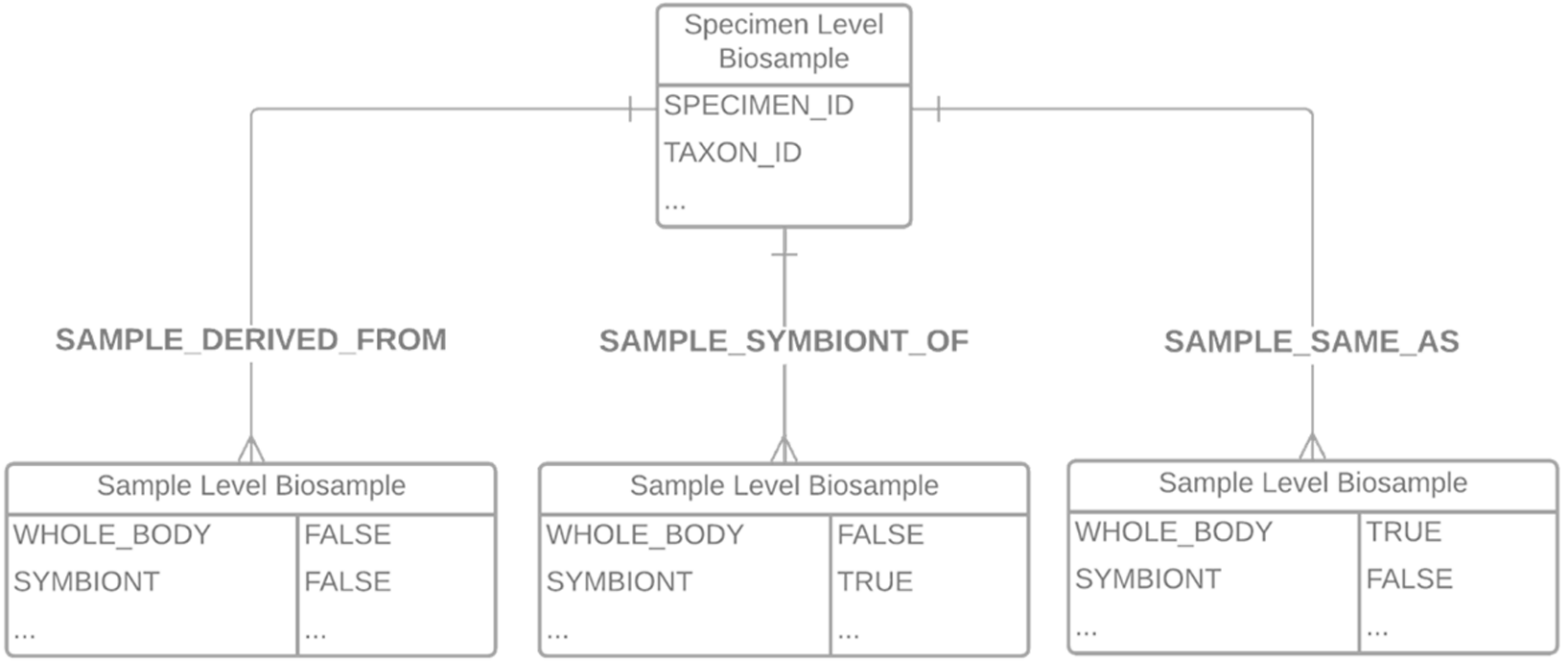
Data model of the relationship between specimens/samples within the BioSample database.

Upon successful validation, COPO sends an email to members of the Sample Supervisors group, to notify of a new manifest awaiting inspection. From this email, a supervisor will navigate to a view within the COPO system, which shows DToL profiles along with their samples. These samples may be filtered according to whether they are pending, accepted or rejected, and their metadata examined for correctness. Pending samples can then be selected either individually or in bundles and accepted or rejected. If accepted, the samples are placed in a queue and are then asynchronously submitted to the ENA for registering the BioSamples. In the background, the sample metadata is converted into a series of XML files required by the ENA to be referenced in subsequent data file uploads. In doing so, we hide significant complexity from the user, thus increasing accessibility and interoperability. Upon successful ingestion, the ENA creates BioSamples for each specimen and for each child sample, according to the data model shown in Figure 2. Within this data model, the individual sample-level BioSamples are linked to the specimen-level BioSample either by a “same as” 1:1 relationship for whole organisms only, a “sample derived from” many:1 relationship for organism parts, or a “sample symbiont of” many:1 relationship for symbionts. This is a simple relationship for single samples, but allows flexibility and granularity when considering more complex relationships, *e.g.* symbionts within a single specimen tube, which has been instrumental in modelling samples from the ASG project. All DToL samples and associated data are linked under ENA study PRJEB40665.

**Figure 3.**
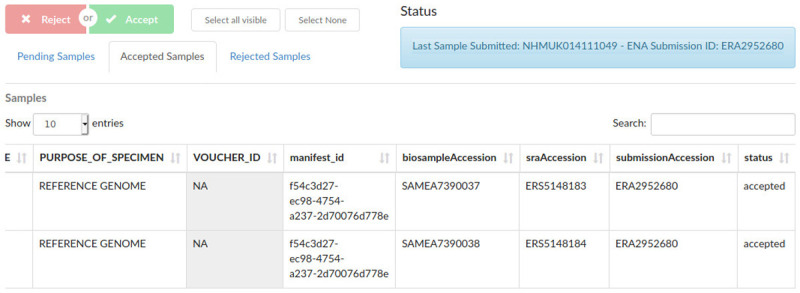
Sample supervisor view, showing accepted sample metadata and European Nucleotide Archive (ENA) and BioSample accession numbers.

Once a specimen-level BioSample has been completely verified, it is allocated an associated public name (ToLID) which uniquely represents the source organism. This is generated by the Sanger Institute based on the species and the SPECIMEN_ID of the sample, and automatically retrieved by COPO during the submission process. The subset of fields that have been identified as relevant for ENA submissions become part of the Biosample metadata. Remaining metadata is kept within COPO and is available through its user and programmatic interfaces. If the submission is successful, each sample, including the “specimen level sample”, is allocated a BioSample accession number that uniquely identifies it in ENA and the BioSample database. COPO then stores these accessions within its database for easy search and retrieval ( Figure 3).

COPO implements an API for collaborators and developers to interact with manifest metadata. The API allows users and other systems to get all manifests, all information about a particular manifest including submission status information, all samples, and all information about a particular sample. In the case of DToL, COPO supplies information to the Genomes on a Tree (GOAT) database ( Sotero-Caio *et al.*, 2021) and STS ( *Tree of Life Sample Management - Wellcome Sanger Institute*, no date), the Sanger Institute Lab Information Management System (LIMS) through these APIs.

More information about ToLIDs and the GOAT database can be found in our sister manuscript ( Lawniczak *et al.*, 2022).

## Imaging

Organism images are an important part of the sample documentation process, and need to be managed alongside metadata. As such, we are currently exploring the mechanisms within COPO with which we can submit images and the key unique identifier information into a suitable public repository. Currently a set of associated sample images can be uploaded. To associate each image with the correct sample metadata in COPO, collectors have to supply image files with names that contain the SPECIMEN_ID with the extension “.png” or “.jpg. Multiple images of the same organism can be submitted by appending an incrementing number to each e.g. “SP123_1.png”, “SP123_2.png”. The final destination of the images is the Bioimage Archive ( ‘The BioImage Archive – Building a Home for Life-Sciences Microscopy Data’, 2022).

## Discussion

We believe we have arrived at a comprehensive set of policies and processes to capture and share rich metadata from a key biodiversity sequencing project which can be useful to others. Our manifests and SOP documents are openly available for other projects to use as a basis for their collection efforts. They represent a common and minimal set of standards, so we would recommend that any projects that wish to use them are free to do so, but any suggestions for changes to the core set of fields and controlled vocabularies should be discussed with the DToL and EBP standards committees. The tools used to collect, validate, and submit this metadata are open source and/or freely available. We actively encourage other biodiversity projects to use these standards and tools and to provide feedback and extensions.

Below we discuss some issues that we experienced along the way so others can factor them into their biodiversity programmes:

### Agreement on headings, valid terms, and ontologies to use for collection standardisation

Engaging with standards development as early as possible in a project is advised. It is inevitable that there will be differences in how a descriptive term represents a piece of information or a process when interacting with individuals or groups of researchers. As such, the field headers used in sample manifests and standardised documentation are vitally important to help researchers understand what information is required to complete the manifest. We engaged with the taxonomic groups over a period of months to ensure that we produced a common set of minimal fields that were easily understandable and clearly partitioned so that metadata collection was as easy as possible, yet still maintained required detail. Arriving at a minimal standard is a useful starting point but can take a large amount of discussion and development, so we would encourage and welcome other projects to use our standard and contact us for advice about potential changes and additions for other biodiversity projects.

### Requirements to support various formats of collection (excel, CSV, phone/tablet app, **among others**)

Whilst many complaints are levelled at spreadsheet programs ( Ziemann *et al.*, 2016), they remain a core tool in data collection for bench and field biologists. Many researchers are taught Excel, or at least learn the basics in school or university courses, and as such are fairly comfortable in its use. DToL needed to ensure the simplest route to collection was provided given that the number of required metadata fields in the manifest is now greater than 50. Whilst samples can be added into COPO through the user interface, this often is not the most useful way to input metadata in batches for larger numbers of samples, so it was agreed that fundamental data collection needed to be supported through spreadsheets.

Other methods of data collection were discussed, *e.g.* phone or tablet apps, and we have not pursued this avenue as yet, but they would be an area for future development. A mobile version of COPO would take a large effort, or at least a manifest completion tool which could then forward on the information via COPO’s APIs, but could be useful to improve uptake.

A future goal is to set up continuous integration (CI) to automatically build new Excel and CSV files and place them in a separate folder in the GitHub repository to act as the definitive spreadsheet version that users can download and use for their sample metadata collection. Maintaining a strict spreadsheet layout and having a single point of reference for downloading and using it can be useful to promote compliance and versioning. It also allows validation at the version level, so submissions made against a previous version can be flagged to a user and the link to the new version can be provided.

### **Validation is often human-**dependent, even with automated mechanisms

Despite all efforts, some human errors cannot be detected and/or fixed by automatic validation. For instance, it was not possible to make sure that the COLLECTION_LOCATION entered was referring to the latest collection ( *e.g.* a museum or an aquarium), and not the original location. In this instance, we actually decided to implement specific fields for each situation to avoid validation problems or subsequent confusion about original versus actual physical collection location. It is often easier to adapt the standard to be unambiguous rather than relying on automated validation to highlight issues. However, changes to standards can result in extensive development time for the systems that implement it, so there is a balance to be made.

Validation can also be affected by unseen modifications in information sources and databases that are outside direct control of a project. For example, we have seen some cases of manifests passing taxonomy validation and subsequently failing soon after, as the taxonomy database is updated often. Therefore, it is vital for large biodiversity programmes to have good dialogue with taxonomic database managers as a result.

### Tools and systems need to be developed closely with specific domains

Generalised tools can be useful in terms of coverage but less so in helping with community engagement and compliance. This is why COPO is developed as an open source and general data brokering platform, but we collaborate with research communities to develop specific brokering routes and user interfaces to match what a given community may expect or need. This can ease the perceived barriers to uptake. Looking to the future, as more sequencing will be carried out at the single-cell level, the importance of accepting sample metadata in plates, subsequent bulk validation, and user interfaces to make this easy to navigate will be required. As LIMS typically have this support, mapping this functionality to the sample collection and data brokering tools should be harmonised.

### Core schemas plus extensions for taxonomic groups can help community uptake, showing that developers are listening to expert collectors

As before, we believe the DToL standards are a good minimal set of well-defined fields and terms for biodiversity projects. However, even within DToL there are corner cases where the core manifest needs extra information which is domain specific, *e.g.* protists. In this case, we worked with the DToL protist groups to develop a handful of extra fields that are supplemental to the core set, *e.g.* salinity, water temperature, pH, among others. These fields can be validated in COPO to accommodate domain-specific requirements but would remain optional in the ToL checklist so when the sample reaches the ENA, it is validated against the core ToL standards and aligned across the project.

### Even with tools and compliance, training and assistance with metadata collection is required

When projects instigate a metadata collection policy, even with the best intentions, a strong connection has to be made and maintained with sample metadata collectors to ensure that documentation is clear, the collection tools are user-friendly, and that people know where to go when errors and issues come up. Using a new software tool for the first time can be daunting, especially when complex information has to be provided. We regularly meet and speak with collectors (virtually through Slack or other online means), and feedback is discussed by the DToL Samples Working Group to understand where changes need to be made to a collection process or metadata standard, or where help can be given to improve uptake or to alleviate pain points.

### Requirements to define update mechanisms for metadata and specifications

Given the size of the project, even with the best efforts and with the most attentive collectors, updates to metadata standards and specifications will always be necessary. A SOP is necessary to define the different scenarios for changes, with respect to important considerations such as regulatory compliance and accountability. Some of these will be actual updates to existing information within the public databases; others will be corrections or clarifications to individual samples or elements of the manifest or SOP itself. Tools and systems also need to be ready to integrate changes, test them, and ensure that updates are propagated to other dependent systems as appropriate. Change management is a vital part of software development to ensure compliance, so this should not be overlooked. As such, looking back to monitor how the manifests have aided uniformity of metadata richness across all submitted samples, as well as compliance to the SOPs and related guidance information, will be useful to inform future development.

When a new metadata upload occurs using updated manifests, it is crucial that the very same validation processes are triggered to avoid inserting metadata that does not respect the original SOP. All of these processes currently result in COPO keeping track of the changes, including the date the metadata was modified and by whom, so it provides an audit of metadata.

## Summary

We have presented some key factors in our efforts to develop a standardised metadata framework, collection procedures, and technical software tools to facilitate the large-scale sample information management for an Earth Biogenome Project sequencing programme. We believe these are useful points of focus for subsequent efforts in this area, and we use our experiences within the UK Darwin Tree of Life project to demonstrate feasibility.

## Data Availability

No data are associated with this article. European Nucleotide Archive: Darwin Tree of Life Project: Genome Data and Assemblies; Accession number: PRJEB40665. https://identifiers.org/ena.embl:PRJEB40665 Zenodo: darwintreeoflife/metadata: Release for Wellcome Open Research, https://doi.org/10.5281/zenodo.7261393 ( Shaw, 2022) Analysis code available from: https://github.com/darwintreeoflife/metadata/tree/v2.4.1 Archived analysis code at time of publication: https://doi.org/10.5281/zenodo.7261393 ( Shaw, 2022) License: MIT
