# Supplementary material for: COPO - Managing sample metadata for biodiversity: considerations from the Darwin Tree of Life project
Source: Wellcome Open Res. 2024 Jun 10;7:279. Originally published 2022 Nov 10. [Version 3] doi: 10.12688/wellcomeopenres.18499.3 (PMC11292180; doi:10.12688/wellcomeopenres.18499.3)
Supplement: Peer review history

# Reviewer report: Reviewer response for version 3

**DOI:** 10.21956/wellcomeopenres.24733.r86233

**Published:** 2024-08-06

**Authors:** Birgitta König-Ries

**Affiliations:** 1 University of Jena, Jena, Germany

I am happy with the changes made and believe the manuscript should be approved now.

Does the article adequately reference differing views and opinions?

Yes

Are all factual statements correct, and are statements and arguments made adequately supported by citations?

Yes

Is the Open Letter written in accessible language?

Yes

Where applicable, are recommendations and next steps explained clearly for others to follow?

Yes

Is the rationale for the Open Letter provided in sufficient detail?

Yes

Reviewer Expertise:

biodiversity informatics

I confirm that I have read this submission and believe that I have an appropriate level of expertise to confirm that it is of an acceptable scientific standard.

# Reviewer report: Reviewer response for version 3

**DOI:** 10.21956/wellcomeopenres.24733.r86232

**Published:** 2024-07-31

**Authors:** Eric Darvish Crandall

**Affiliations:** 1 The Pennsylvania State University - University Park Campus, University Park, Pennsylvania, USA

Thank you for the revisions to the API that will allow export to the various biodiversity and genetic standards, for clarifying about the checklist and for citing GEOME. I approve the new version of this manuscript.

Does the article adequately reference differing views and opinions?

No

Are all factual statements correct, and are statements and arguments made adequately supported by citations?

Yes

Is the Open Letter written in accessible language?

Yes

Where applicable, are recommendations and next steps explained clearly for others to follow?

Yes

Is the rationale for the Open Letter provided in sufficient detail?

Yes

Reviewer Expertise:

Population genetics and genomics, Genomic Metadata, Open Science

I confirm that I have read this submission and believe that I have an appropriate level of expertise to confirm that it is of an acceptable scientific standard.

# Reviewer report: Reviewer response for version 2

**DOI:** 10.21956/wellcomeopenres.21928.r72317

**Published:** 2024-01-05

**Authors:** Eric Darvish Crandall

**Affiliations:** 1 The Pennsylvania State University - University Park Campus, University Park, Pennsylvania, USA

The authors describe metadata standards, a minimum information checklist and metadata brokering software (Collaborative Open Genomics; COPO) developed for the Darwin Tree of Life project. The standard, checklist and software are described as a contribution to making genomic datasets more findable, accessible, interoperable and reusable (FAIR).

 Overall, I am glad to find like-minded researchers in Europe working toward a common goal of FAIR and open genomic data. I appreciate the care given to metadata curation, especially via Sample Supervisors. In full disclosure (see also my conflict of interest statement), I sit on the steering committee for the Genomic Observatories Metadatabase, (https://geome-db.org; see Deck *et al.*, 2017 ^1^, Riginos *et al.*, 2020 ^2^, Toczydlowski *et al.*, 2021 ^3^ and Crandall *et al.*, 2023 ^4^).

 I have some significant reservations about implementation as described in the current version of this letter.

1) New metadata standard seems redundant:

 As with all academic endeavors, it is important to acknowledge and build upon previous work. It is a little unclear whether the authors are proposing a new standard or merely new software, but I will take the following sentence from the abstract as evidence of the former: "Here we report on the standards we developed with respect to a robust and reusable mechanism of metadata collection, in the hopes that other projects forthcoming or underway will adopt these practices for metadata."

 In the first paragraph of the section entitled Sample Manifest Development, the authors mention existing standards that govern biodiversity data and genomic data: Darwin Core and MIxS respectively. In the next paragraph they already depart from these standards, using SPECIMEN_ID rather than materialSampleID (Darwin Core) or source_mat_id (MIxS) for the all important sample identifier field. Actually, as I peruse the linked DToL_SAMPLE_MANIFEST_v2.4.xlsx, most of the fields do not match with Darwin Core or MIxS. For example, there is DEPTH instead of maximumDepthInMeters and minimumDepthInMeters (DarwinCore). Similarly with ELEVATION vs. minimumElevationInMeters and maximumElevationInMeters (Darwin Core). I suppose SCIENTIFIC_NAME is supposed to match up with specificEpithet (Darwin Core), since GENUS is a separate term, but it is unclear, as there are no definitions given in the manifest. Instead of yearCollected, monthCollected, dayCollected recommended by Darwin Core to avoid common ambiguities in dates, there is DATE_OF_COLLECTION, with a note to keep data in YYYY-MM-DD format (but we know that validation can't always be applied and some users will forget). There are others like DECIMAL_LATITUDE instead of decimalLatitude, that seem innocuous but could still impact data findability and interoperability. Similarly, TUBE_OR_WELL_ID would better be samp_well_name (MIxS), etc.

 As the authors state in the discussion: "... the field headers used in sample manifests and standardised documentation are vitally important to help researchers understand what information is required to complete the manifest." Departing from existing standards defeats their attempt to make their data, and that of future users (ERGA and ASG are given as examples) findable, accessible, interoperable and reusable (FAIR). At the end of the section, there is a note about mapping terms to Darwin Core, but why not just use the Darwin Core terms in COPO directly to avoid any possibility of ambiguity? At GEOME we have found that neither standard covers all of the terms that we need, so we use a blend of the two, always providing clear definitions in the metadata template (our equivalent of the sample manifest). We only create new terms when it is clear that neither standard has a term that covers our needs.

 I worry that creating a new metadata standard for genomic data where (admittedly imperfect) standards already exist will only add to the confusion around genomic metadata. For example, imagine trying to merge a COPO dataset with one found on GBIF (which uses Darwin Core) or the SRA (which is adopting MIxS). The GSC and TDWG have recently agreed to map their similar terms to one another's standard (http://www.gensc.org//news/2022/11/04/gsc_tdwg_mou.html), thereby reducing confusion around their overlap. I'd prefer if the authors not increase confusion again by creating new redundant terms for COPO and its users, and instead adhere to Darwin Core and MIxS whenever possible.

 The authors provide examples of mapping their terms to Darwin Core, but nowhere is a complete mapping given. And why not just use accepted terms in the first place instead of creating a redundant standard?

 It is this important reservation that causes me to not approve the letter.

2) Checklist alignment:

 Reasonable people can disagree about what metadata should comprise a minimum set for a genomic dataset. In fact I have found that each time I engage in this exercise I arrive at a different answer, depending on the context of the data and who is part of the conversation. The authors provide another such list with the statement:

 "The checklist also aligns the metadata collected for the DToL manifest against other existing standards (for example the MIxS standards (Yilmaz et al., 2011)) to allow comparison with samples outside of the project."

 I see no evidence of this alignment in the linked checklist (at https://www.ebi.ac.uk/ena/browser/view/ERC000053). Moreover, the minimum information requirements (i.e. which terms are required, recommended, optional) differ significantly from the minimums defined by the Genomics Standards Consortium via MIxS (http://www.gensc.org/pages/standards/checklists.html). There should at least be an explanation for the differences, otherwise why should this checklist prevail over any other?

3) Citations to GEOME:

 In a more commercial setting, GEOME and COPO might be considered competitors, but I hope that in an academic tradition we can be collaborators, both working towards FAIR genomic data. I think we ultimately want to both use our slightly different platforms to arrive at this same goal. I hope my comments can be taken in this spirit. I will seek to cite COPO going forward (and have already done so in a manuscript in review), and I hope this courtesy can be returned.

Does the article adequately reference differing views and opinions?

No

Are all factual statements correct, and are statements and arguments made adequately supported by citations?

Yes

Is the Open Letter written in accessible language?

Yes

Where applicable, are recommendations and next steps explained clearly for others to follow?

Yes

Is the rationale for the Open Letter provided in sufficient detail?

Yes

Reviewer Expertise:

Population genetics and genomics, Genomic Metadata, Open Science

I confirm that I have read this submission and believe that I have an appropriate level of expertise to state that I do not consider it to be of an acceptable scientific standard, for reasons outlined above.

## References

[1] : The Genomic Observatories Metadatabase (GeOMe): A new repository for field and sampling event metadata associated with genetic samples. *PLoS Biol* .2017;15(8) : 10.1371/journal.pbio.2002925 e2002925 10.1371/journal.pbio.2002925 28771471 PMC5542426

[2] : Building a global genomics observatory: Using GEOME (the Genomic Observatories Metadatabase) to expedite and improve deposition and retrieval of genetic data and metadata for biodiversity research. *Mol Ecol Resour* .2020;20(6) : 10.1111/1755-0998.13269 1458-1469 10.1111/1755-0998.13269 33031625

[3] : Poor data stewardship will hinder global genetic diversity surveillance. *Proc Natl Acad Sci U S A* .2021;118(34) : 10.1073/pnas.2107934118 10.1073/pnas.2107934118 34404731 PMC8403888

[4] : Importance of timely metadata curation to the global surveillance of genetic diversity. *Conserv Biol* .2023;37(4) : 10.1111/cobi.14061 e14061 10.1111/cobi.14061 36704891 PMC10751740

# Response

**Published:** 2024-05-13

**Authors:** Felix Shaw

**Reviewer Comment:**

 Point 1: “As with all academic endeavors, it is important to acknowledge and build upon previous work. It is a little unclear whether the authors are proposing a new standard or merely new software, but I will take the following sentence from the abstract as evidence of the former: "Here we report on the standards we developed with respect to a robust and reusable mechanism of metadata collection, in the hopes that other projects forthcoming or underway will adopt these practices for metadata."  In the first paragraph of the section entitled Sample Manifest Development, the authors mention existing standards that govern biodiversity data and genomic data: Darwin Core and MIxS respectively. In the next paragraph they already depart from these standards, using SPECIMEN_ID rather than materialSampleID (Darwin Core) or source_mat_id (MIxS) for the all important sample identifier field. Actually, as I peruse the linked DToL_SAMPLE_MANIFEST_v2.4.xlsx, most of the fields do not match with Darwin Core or MIxS. For example, there is DEPTH instead of maximumDepthInMeters and minimumDepthInMeters (DarwinCore). Similarly with ELEVATION vs. minimumElevationInMeters and maximumElevationInMeters (Darwin Core). I suppose SCIENTIFIC_NAME is supposed to match up with specificEpithet (Darwin Core), since GENUS is a separate term, but it is unclear, as there are no definitions given in the manifest. Instead of yearCollected, monthCollected, dayCollected recommended by Darwin Core to avoid common ambiguities in dates, there is DATE_OF_COLLECTION, with a note to keep data in YYYY-MM-DD format (but we know that validation can't always be applied and some users will forget). There are others like DECIMAL_LATITUDE instead of decimalLatitude, that seem innocuous but could still impact data findability and interoperability. Similarly, TUBE_OR_WELL_ID would better be samp_well_name (MIxS), etc. As the authors state in the discussion: "... the field headers used in sample manifests and standardised documentation are vitally important to help researchers understand what information is required to complete the manifest." Departing from existing standards defeats their attempt to make their data, and that of future users (ERGA and ASG are given as examples) findable, accessible, interoperable and reusable (FAIR). At the end of the section, there is a note about mapping terms to Darwin Core, but why not just use the Darwin Core terms in COPO directly to avoid any possibility of ambiguity? At GEOME we have found that neither standard covers all of the terms that we need, so we use a blend of the two, always providing clear definitions in the metadata template (our equivalent of the sample manifest). We only create new terms when it is clear that neither standard has a term that covers our needs. I worry that creating a new metadata standard for genomic data where (admittedly imperfect) standards already exist will only add to the confusion around genomic metadata. For example, imagine trying to merge a COPO dataset with one found on GBIF (which uses Darwin Core) or the SRA (which is adopting MIxS). The GSC and TDWG have recently agreed to map their similar terms to one another's standard ( http://www.gensc.org//news/2022/11/04/gsc_tdwg_mou.html), thereby reducing confusion around their overlap. I'd prefer if the authors not increase confusion again by creating new redundant terms for COPO and its users, and instead adhere to Darwin Core and MIxS whenever possible. The authors provide examples of mapping their terms to Darwin Core, but nowhere is a complete mapping given. And why not just use accepted terms in the first place instead of creating a redundant standard? It is this important reservation that causes me to not approve the letter.”

***Author Response:***

 We appreciate the thoughtful feedback and acknowledge the concerns raised regarding the departure from existing standards in the initial stages of metadata development. In response to these concerns, we would like to offer the following rebuttal:

- Domain Specificity:
  - Darwin Tree of Life’s metadata schema is designed from the bottom up, for its intended use case, mass bioscreening and biocuration. Therefore the field names were carefully customised by the Sample Working Group. It is not within COPO's remit to impose field names on the community. We instead take input from the community on how they would like to approach metadata collection most effectively for their project (in this case the generation of the sample manifest) then we make efforts, alongside the ENA to align the terminology and validation of the fields to existing standards to ensure interoperability. As the reviewer suggests, we do map our fields to existing standards, thus enabling the interoperability of DToL sample metadata with the community at large. COPO’s API now allows any of the data collected to be exported to any of the commonly used standards, and in a variety of textual formats such as json, csv and ROCrate.
- Mapping to Darwin Core and MIxS:
  - Our metadata now aligns where possible, to MIxS and DWC, these mappings are available in the API. We acknowledge the importance of alignment with existing standards for the sake of interoperability and to ease potential integration with other datasets. The ongoing efforts to map our terms to Darwin Core reflect our commitment to harmonising our metadata schema with established standards.
- Immediate Benefits and Long-Term Alignment:
  - The decision to deviate from existing standards initially was driven by the needs of the community, and the immediate need to start collecting data efficiently. We assure the reviewer that, as the project progresses, we are dedicated to aligning our metadata schema closely with Darwin Core and MIxS, ensuring long-term compatibility and adherence to widely accepted practices. Whilst this is an ongoing and imperfect process, we strongly believe that a working solution to 90% of the problem is better than a “perfect” solution which never reaches fruition.
- COPO's Adaptive Export Functionality:
  - We highlight that COPO's API is designed to facilitate data export in various standards, which now supports MIxS and Darwin Core. This means that while the initial data collection may use a specific schema, researchers can export data in formats that align with widely accepted standards, mitigating potential interoperability issues.
- Openness to Community Feedback:
  - We express our openness to feedback and collaborative input from the scientific community. We value the concerns raised and view them as valuable insights that can contribute to the ongoing refinement of our metadata schema. The iterative nature of the project allows for continuous improvement based on feedback and emerging best practices.

  In addition to the points mentioned above, we would like to emphasise the following aspects regarding COPO's role as a data broker and our philosophy on standards development:

- COPO as a Data Broker:
  - Our primary goal with COPO is to act as a data broker, facilitating seamless exchange and management of research data. Recognising the diverse needs and workflows of researchers, we aim to serve as an intermediary that eases the burden on researchers during data collection, curation, and sharing processes.
- Bottom-Up Standards Development:
  - We strongly advocate for a bottom-up approach to standards development. We believe that standards should evolve organically from the research community, taking into account the specific needs and practices of individual researchers and projects. COPO's design reflects this philosophy, empowering researchers to contribute to the development of standards based on their unique requirements and preferences. It is then our job to ensure data alignment.
- Easing the Burden on Researchers:
  - COPO is designed to alleviate the challenges faced by researchers in adopting and adhering to standards. Our platform is user-friendly and adaptable, recognising the dynamic nature of research projects. By allowing researchers to work with familiar terms and map them to established standards, we aim to strike a balance between flexibility and adherence to best practices.
- Continuous Improvement and Community Involvement:
  - We are committed to continuous improvement based on community feedback. COPO's development is an ongoing process, and we invite researchers to actively engage with COPO, share their experiences, and contribute to the platform's evolution. This collaborative approach ensures that COPO remains responsive to the evolving needs of the research community.

**Reviewer Comment:**

 Point 2:  “Reasonable people can disagree about what metadata should comprise a minimum set for a genomic dataset. In fact I have found that each time I engage in this exercise I arrive at a different answer, depending on the context of the data and who is part of the conversation. The authors provide another such list with the statement:   "The checklist also aligns the metadata collected for the DToL manifest against other existing standards (for example the MIxS standards (Yilmaz et al., 2011)) to allow comparison with samples outside of the project."   I see no evidence of this alignment in the linked checklist (at https://www.ebi.ac.uk/ena/browser/view/ERC000053). Moreover, the minimum information requirements (i.e. which terms are required, recommended, optional) differ significantly from the minimums defined by the Genomics Standards Consortium via MIxS ( http://www.gensc.org/pages/standards/checklists.html). There should at least be an explanation for the differences, otherwise why should this checklist prevail over any other?”

***Author Response:***

 Thank you for your comments and for raising these concerns regarding the ENA checklist alignment to standards. We understand that we have not been clear in describing this alignment. As mentioned in the manuscript, the ENA sample checklists capture the requirements on the minimum metadata needed to describe biological samples. The ENA holds a number of different checklists, including the full set of MIxS environmental extensions to core packages, that are currently being updated to version 6.2. In the majority of cases, the MIxS terms are added as specified by the GSC, however sometimes there needs to be some slight adjustments to (a) fit the ENA data model (e.g. some terms such as the sequencing method are directly associated to the data and not to the sample, therefore this may not be mandatory for sample submission) or (b) if another standard already uses a pre-defined term for the same use, efforts are made to align these to enhance interoperability across the use in different ENA checklists.  As mentioned above, as the ToL metadata schema was designed bottom-up to respond to the specific needs of the community, it is not totally compliant with MixS. The ENA Tree of Life Checklist was designed to validate the ToL sample metadata with terminology aligned to existing ENA submitting standards, including the ENA implementation of the MIxS standards to allow interoperability. We have corrected the sentence in the text so it reflects more accurately what has been done for the ToL checklist. It now reads “The terminology used in the checklist also aligns syntactically the metadata collected for the DToL manifest against other existing standards (including the MIxS standards ( Yilmaz et al., 2011)) to allow comparison with samples outside of the project.”

**Reviewer Comment:**

 Point 3: “In a more commercial setting, GEOME and COPO might be considered competitors, but I hope that in an academic tradition we can be collaborators, both working towards FAIR genomic data. I think we ultimately want to both use our slightly different platforms to arrive at this same goal. I hope my comments can be taken in this spirit. I will seek to cite COPO going forward (and have already done so in a manuscript in review), and I hope this courtesy can be returned”.

***Author Response:***

 Thank you for your thoughtful comments, and we genuinely appreciate the insights you provided. We acknowledge the oversight in omitting a reference to GEOME in our initial manuscript and have addressed this in the resubmitted version. We are pleased to learn about the significant work carried out by GEOME and fully concur with your perspective on collaboration in academic endeavours. The shared objective of advancing FAIR genomic data is paramount, and we believe that the collaboration of GEOME and COPO can contribute meaningfully to this mission. We are enthusiastic about the potential for collaboration and sincerely thank you for your openness to such an engagement. Moving forward, we commit to citing GEOME in our future work, and we hope for a similar consideration in return. Your positive approach resonates with our mission, and we anticipate fruitful joint efforts in advancing genomic data standards. Your constructive feedback is invaluable, and we deeply appreciate your contribution to our shared goals.

# Reviewer report: Reviewer response for version 2

**DOI:** 10.21956/wellcomeopenres.21928.r64298

**Published:** 2023-08-01

**Authors:** Katrina Exter

**Affiliations:** 1 Vlaams Instituut voor de Zee, Ostend, Flanders, Belgium

No further comments.

Does the article adequately reference differing views and opinions?

Yes

Are all factual statements correct, and are statements and arguments made adequately supported by citations?

Yes

Is the Open Letter written in accessible language?

Yes

Where applicable, are recommendations and next steps explained clearly for others to follow?

Yes

Is the rationale for the Open Letter provided in sufficient detail?

Yes

Reviewer Expertise:

Open science; data managment; marine biodiversity

I confirm that I have read this submission and believe that I have an appropriate level of expertise to confirm that it is of an acceptable scientific standard.

# Reviewer report: Reviewer response for version 1

**DOI:** 10.21956/wellcomeopenres.20513.r57911

**Published:** 2023-07-12

**Authors:** Birgitta König-Ries

**Affiliations:** 1 University of Jena, Jena, Germany

This article describes the developments towards standardisation, collection and preservation of metadata describing the samples that form the basis for sequencing in the Darwin Tree of Life project. I believe this to be a valuable resource for people involved in this project in some way, interested in working with the data or aiming to set up similar projects (on the same or even on smaller scales). 

 In my opinion, the paper is very definitely worth to be indexed.

 I believe, though, that it would profit from some reorganisation. I found the flow of information not always easy to follow and had the impression that different threads that could well be separated were intertwined in the story line. 

 To me, the description of the metadata standard, the SOPs, and so on (so basically the artefacts that were developed in the project to support metadata management) should be described separately from the workflow used when entering a sample into the system and adding metadata to it. For this workflow description, an overview figure would be very helpful.

 Some more detailed remarks:

**Introduction**

- paragraph 2: "providing human understanding": A major aim of FAIR data and metadata is to provide not only human but also machine understanding (or machine actionability or whatever you want to call it). I assume that is also what you aim for here.
- paragraph 3: "Therefore, standardised and .... " That list seems more exemplary than complete. If that is true you may want to rephrase. 

**Sample manifest development**

- paragraph 2, 3rd line from the bottom: 
  - RACK_OR_PLATE_ID and TUPE_OR_WELL_ID: From the text it is not quite clear whether that and means one of the two or both

**page 4, left column, bottom paragraph: **

- Are the different versions of the manifest developed in the google docs persisted somewhere and can be referred to via a persistent identifier. If not: Why is that not needed?
- If manifests or SOPs change: What happens to metadata/samples processed and described with older versions? Is there some reprocessing?

**page 7, figure 2: **

- A legend needs to be provided and/or some standard form of diagram (ER, UML Class, ...) should be used. 

**page 7. left column, first paragraph: **

- "names that contain...": Does that mean there is only one image file per specimen possible or can the filenames contain additional information. If so: how is that standardized to ensure interpretabilitiy?

Does the article adequately reference differing views and opinions?

Yes

Are all factual statements correct, and are statements and arguments made adequately supported by citations?

Yes

Is the Open Letter written in accessible language?

Yes

Where applicable, are recommendations and next steps explained clearly for others to follow?

Yes

Is the rationale for the Open Letter provided in sufficient detail?

Yes

Reviewer Expertise:

research data management, biodiversity informatics

I confirm that I have read this submission and believe that I have an appropriate level of expertise to confirm that it is of an acceptable scientific standard, however I have significant reservations, as outlined above.

# Response

**Published:** 2023-07-25

**Authors:** Felix Shaw

I thank the reviewer for their time and thoughtful criticism. I have taken on board these and have altered the manuscript, with some rewording where things were not clear, clarifications and additions where asked for and a refactor of the figure of the COPO/Biosamples data model (figure 2).

# Reviewer report: Reviewer response for version 1

**DOI:** 10.21956/wellcomeopenres.20513.r59555

**Published:** 2023-06-14

**Authors:** Katrina Exter

**Affiliations:** 1 Vlaams Instituut voor de Zee, Ostend, Flanders, Belgium

Making the data and knowledge arising from genomics-based biodiversity studies fully FAIR is a vital endeavour. The importance of these studies to our understanding of biodiversity loss, and how to mitigate that loss, is enormous: smoothing the pathway to the rapid uptake of the results of these studies, and making it easier for the outputs from the numerous studies taking place around the world to be meaningfully combined and compared, is crucial. FAIR data management, starting from the field scientists collecting the data and continuing through to the publication and re-use of the data, is a necessary for any of this to happen. Emphasising the interoperability, understandability, and provenance of these data, is key. 

 The DToL project will produce extremely useful data and science for genomics, biodiversity, and taxonomy specialists. Explaining how the data and metadata are managed by DToL is important: (1) it explains how the data are created, standardised, and shared, creating trust in the project’s outputs, (2) it contributes to the world-wide efforts that are being made to do FAIR data management. This paper, together with its related paper quoted therein, gives a good overview of the approach taken with the management of the metadata collected from the field scientists and curated by the DToL, and the submission of the (meta)data to ENA. It explains the steps undertaken, and very usefully shares the lessons learned. Links are given to their GitHub repository where the reader can investigate the metadata SOP and template in more detail; the software used are open source: taken together, the same methodology could be ad(o)(a)pted by others. 

 The formatting and standardisation of the sample metadata (the logsheets) are well described: fixed columns and formatted entries are required. One comment I would add here is that these metadata would be even more interoperable (especially to machines/developers) if more semantics were added. There *is* a short paragraph in the text about “mapping” the column names to terms from controlled vocabularies, and when this is complete I would recommend that this mapping file is also made available in GitHub. This would also help with backward compatibility (it was mentioned that column titles can change or be added). Where terms cannot be found, they could be created within a DToL vocabulary. Exposing these now semantically-annotated metadata as RDF would go even one step further in making the metadata and the described data more usable by others. 

 Another comment I have is that it would also have been useful (especially for the data-specialist readers) to have included a diagram of the DToL (meta)data model.

 Looking into more detail of the (meta)data, I have a few suggestions to the project 

- It would improve the machine-interoperability and tracking of the SOP(s) if it was described in a machine-accessible way. This is not common practice, but nonetheless useful to do: see https://github.com/BeBOP-OBON for one such approach.  
- With respect to the terminology used for indicating which parts of the body samples were taken from: have DToL considered adopting (or creating) an ontology here?
- Information about the SOPs and/or standardisation of the sample preparation and sequencing is missing: no link to a current or future article where this is explained is given. As sample preparation can have an impact on the sequences subsequently obtained, and the methodology of sequencing is even now still an evolving field, the steps taken to ensure the provenance of the genomics results are important to share. This does not detract from this article, however I would recommend that DToL considers such a publication. 

** **


****

Does the article adequately reference differing views and opinions?

Yes

Are all factual statements correct, and are statements and arguments made adequately supported by citations?

Yes

Is the Open Letter written in accessible language?

Yes

Where applicable, are recommendations and next steps explained clearly for others to follow?

Yes

Is the rationale for the Open Letter provided in sufficient detail?

Yes

Reviewer Expertise:

Open science; data managment; marine biodiversity

I confirm that I have read this submission and believe that I have an appropriate level of expertise to confirm that it is of an acceptable scientific standard.

# Response

**Published:** 2023-07-25

**Authors:** Felix Shaw

I thank the reviewer for their valuable insight and time in reviewing this article. With regards to the specific points raised, the details regarding sequencing are published in genome notes very regularly on wellcome open research, and I believe these contain the particulars e.g. https://wellcomeopenresearch.org/articles/8-319/v1. With regards to this paper, we are talking about metadata, so the specifics of genomic sequencing and library prep are out of scope. An ontology for the body parts was not considered, but I agree this would be a step in the right direction. I will look at the BeBOP-OBON link with interest. We are currently looking at ROCrate as a way of making outputs from COPO's API more machine readable (https://www.researchobject.org/ro-crate/).
